# Treatment Strategy for Currarino Syndrome Complicated With Anorectal Stenosis

**DOI:** 10.7759/cureus.50512

**Published:** 2023-12-14

**Authors:** Atsushi Harada, Hirofumi Tomita, Ayano Tsukizaki, Yuki Mizuno, Hideo Ishihama, Akihiro Shimotakahara, Kentaro Matsuoka, Naoki Shimojima, Seiichi Hirobe

**Affiliations:** 1 Surgery, Tokyo Metropolitan Children’s Medical Center, Tokyo, JPN; 2 Pathology, Tokyo Metropolitan Children’s Medical Center, Tokyo, JPN

**Keywords:** anorectal malformation, currarino triad, tethered spinal cord, meningocele, sacrococcygeal teratoma

## Abstract

Purpose: The present study aimed to review the treatment experience and outcomes of Currarino syndrome (CS) complicated with anorectal stenosis to evaluate the current treatment strategies.

Methods: Seven cases of CS complicated with anorectal stenosis, treated at our hospital between 1998 and 2021, were retrospectively investigated. This is a case series article from a single institution.

Results: In six and three cases and one case, the presacral mass was a mature teratoma, meningocele, and lipoma, respectively. Resection of the lesion was performed in all six cases of mature teratoma, and duraplasty was performed before resection in all three cases of meningocele. Moreover, surgery for anorectal stenosis was performed simultaneously in four patients. Surgery was performed for six cases of anorectal stenosis, with the remaining case relieved by dilation using a metal bougie. The surgical methods used were a partial resection with end-to-end anastomosis, anorectal strictureplasty, pull-through, posterior sagittal anorectoplasty, and cutback after mass resection. Pathological analysis of the anorectal stenoses revealed disorganized and rough smooth muscle fibers and the replacement of the stroma by an increased quantity of collagen fibers.

Conclusions: The clinical outcomes of CS can be improved by establishing a treatment flow chart and understanding the complicated pathophysiology of the disease.

## Introduction

Currarino syndrome (CS) is a rare disease with an incidence of approximately one in 100,000 [1], characterized by three clinical features: presacral mass, anorectal malformation, and sacral bone deformity. Some variants of this “Currarino triad” require not only pediatric surgery but also neurosurgery. The most common type of anorectal malformation associated with CS is anorectal stenosis [2], whose severity and extent vary.

There are currently no established guidelines on CS treatment owing to the paucity of comprehensive studies of the disease [1,3]. We herein reviewed the clinical course, surgical methods, and outcomes of CS cases treated at our hospital and retrospectively suggested strategies for the treatment of CS associated with anorectal stenosis because there are a few previous reports [1,3] that clearly summarize the order of surgical treatment and determined surgical methods for anorectal stenosis according to clinical symptoms.

## Materials and methods

The present, retrospective review of the records of patients with Currarino syndrome (CS) who underwent surgery for the disease was approved by the Institutional Review Board of Tokyo Metropolitan Children’s Medical Center. The present study was approved by the Institutional Review Board (Research Ethics Review Board No. 2020b-21).

Seven patients with CS with anorectal stenosis who were seen at our hospital between 1998 and 2021 were reviewed for the clinical detail, primary symptoms, histological findings of any presacral mass, medical history of intra-tumoral infection, presence of a fistula between the presacral mass and anorectum, level of sacral bone deformity, associated malformations, family history of CS, surgical method, order for treatments for anorectal stenosis, presacral mass, and spinal tethered cord, pathological findings of anorectal stenosis, and postoperative defecation function.

The diagnosis of these patients with CS was performed based on a triad of sacral malformation, presacral tumor, and anorectal stenosis. All patients were diagnosed using ultrasonography (US) and magnetic resonance imaging (MRI). Computed tomography (CT) and contrasted enema were additionally performed for some patients for the evaluation of anorectal stenosis. Familial history included even in the cases of incomplete CS.

Histological evaluation was evaluated with hematoxylin and eosin (H-E) staining and azan staining using the standard protocol. The presence of a fistula between the presacral mass and anorectum was evaluated from the histopathological findings in the presacral mass and specimens of anorectal stenosis.

## Results

Patient background

All seven patients with CS with associated anorectal stenosis treated at our hospital were subadult females. Their median age at CS diagnosis was eight months (range: 1-18 months). The primary symptoms were abdominal distension in three patients, constipation in two patients, sacral skin depression and urinary tract infection in one patient, and anuresis in one patient. Pathological analysis of the presacral masses found a mature teratoma in six patients; a meningocele in three patients; and a lipoma, including duplication, in one patient. Three patients had a fistula communicating between the tumor and anorectum, and two of these three patients had a past medical history of intratumoral infection. All cases of sacral bone deformity were scimitar-type sacral bone defects, with three and four cases of S3- and S4-level sacral bone defects, respectively. Five cases of the tethered spinal cord were observed. Other, associated, congenital malformations were scoliosis or a giant ureter in one patient and an anterior anus in two patients. Table 1 summarizes the details of each patient.

**Table 1:** Summary of patient characteristics, clinical course, radiological operation of seven cases with Currarino syndrome. F: Female UTI: urinary tract infection, PSARP: posterior sagittal anorectal plasty, SSI: surgical site infection, m: month, y: year

Case	Sex	Primary symptom	Age	Preoperative defecation function	Presacral mass	History of infection	Fistula with presacral mass	Sacral bone defect	Family history	Tethered cord	Surgical treatment	Complications after surgery
1	F	Abdominal distension	1m	Continuous transanal drainage	Mature teratoma, meningocele	No	No	S3	No	No	1m: Colostomy 1y1m: Duraplasty 1y6m: Resection of tumor and the lesion in anorectal stenosis 1y10m: Stoma closure	Neurogenic bladder
2	F	Constipation	1y3m	Glycerin enema with laxative, Bougie dilation	Lipoma	No	No	S4	No	Yes	1y10m: Untethering 2y3m: PSARP	Fecal impaction
3	F	Abdominal distension	1y	Glycerin enema with laxative, Bougie dilation	Mature teratoma	No	No	S4	No	No	1y4m: Tumor resection, strictureplasty	None
4	F	Sacral skin depression	4m	None	Mature teratoma	No	No	S4	Yes	Yes	4m: Untethering, anal bougie 2y9m: Tumor resection	None
5	F	Abdominal distention, anuresis	7m	None	Mature teratoma	Yes	Yes	S4	Yes	Yes	9m: Untethering, tumor resection 1y7m: Cutback	SSI， Fecal impaction
6	F	UTI	8m	Glycerin enema	Mature teratoma, meningocele	Yes	Yes	S3	No	Yes	9m: Untethering, duraplasty 11m: Tumor resection, strictureplasty, covering ileostomy 1y3m: Stoma closure	SSI
7	F	Chronic constipation	1y6m	Glycerin enema, Bougie dilation	Mature teratoma, meningocele	No	Yes	S3	Yes	Yes	1y10m: Untethering duraplasty 2y4m: Tumor resection, colon resection, Soave-Denda pull-through, covering ileostomy 2y6m: Stoma closure	None

Neurosurgery for CS

The order of neurosurgery for CS varied by patients, as seen in Table 1. All five patients with tethered spinal cords underwent untethering surgery simultaneously or before surgery for anorectal stenosis and presacral mass resection. All three patients with a presacral meningocele also had an associated teratoma. Duraplasty was required in these patients to reduce the risk of meningitis because the presacral mass was complicated with a fistula communicating the intraspinal cavity with the anorectum. In two of the three patients, the operation was performed simultaneously with untethering surgery.

Surgical treatment for the anorectal stenosis and presacral mass

Only one patient (Case 1) required a colostomy for severe anorectal stenosis. Surgery was performed for anorectal stenosis in six patients, except the patient in Case 4, who underwent dilation with a metal bougie. The surgical method for anorectal stenosis treatment was end-to-end anastomosis with a partial resection, anorectal strictureplasty, pull-through, posterior sagittal anorectoplasty (PSARP), and cutback after presacral mass resection. In four patients, the surgery for anorectal stenosis was performed simultaneously with excision of the presacral mass. Surgical resection of the presacral mass was performed in all six patients with a mature teratoma.

In Case 2, PSARP was performed because the anterior anus had a moderate degree of stenosis, and the presacral mass was diagnosed as a lipoma based on radiological findings and, therefore, did not require removal. In Case 5, stenosis of the anterior anus was initially diagnosed as mild stenosis not requiring surgery; however, a cutback procedure involving transanal excision of scar tissue was performed to relieve the stenosis after severe fecal embolization requiring disimpaction occurred after resection of the infected presacral mass. In Case 7, a pull-through procedure was performed for the anorectal stenosis according to the Soave-Denda method, which is typically performed for Hirschsprung’s disease. In Cases 6 and 7, a covering ileostomy was created simultaneously with the surgery for anorectal stenosis to reduce the risk of anastomotic leakage.

Postoperative course and defecation/urinary function

In terms of complications in the early postoperative period, two patients in Cases 5 and 6 had a deep surgical site infection; both had a history of a preoperative intratumoral infection, which was treated with lavage. The current, median age of the patients is seven years (range: 2-23 years), and the median follow-up period from the last surgery is five years (range: 1-21 years). In terms of long-term, postoperative defecation function, two patients required hospitalization for fecal impaction, and a cutback was performed additionally for the anorectal stenosis in Case 5, as described above. Except for Case 4, which was treated only with anal dilation with a metal bougie, glycerin enemas were required for defecation management owing to constipation, but none of the patients experienced incontinence. To restore postoperative urinary function, clean intermittent catheterization was begun for neurogenic bladder in the patient in Case 1 at age 15 years. The other patients did not experience any urinary issues.

Pathological findings of anorectal stenosis in resected specimens

**Figure 1:**
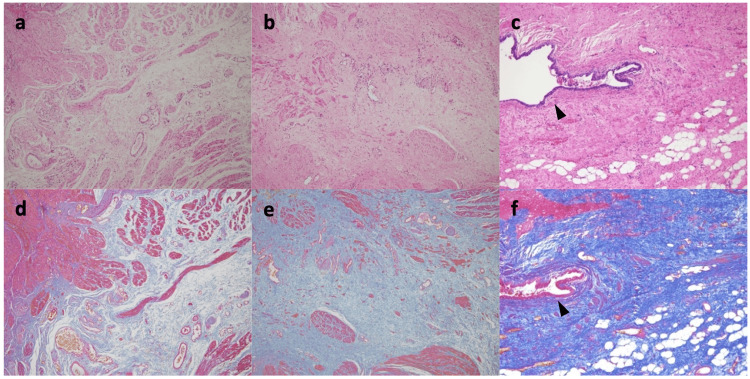
Pathological findings of the anorectal stenosis. Figure 1 (a, d) Case 1 and (b, e) Case 2. Pathological analysis of the anorectal stenoses revealed disorganized and rough smooth muscle fibers and replacement of the stroma by an increased quantity of collagen fibers (20x magnification). Figure 1 (c, f) Case 7. Pathological analysis of the anorectal stenosis revealed disorganized and rough smooth muscle fibers and replacement of the stroma by an increased quantity of collagen fibers. In addition, the fistula communicating the anorectum with the tumor was seen, which histopathological analysis revealed to be lined with columnar or squamous epithelium (black arrow, 20x magnification). Figure 1 (a, b, c) Hematoxylin-eosin stain; (d, e, f) Azan stain.

Resected specimens of the anorectal stenosis were obtained from five patients (Cases 1, 2, 5, 6, and 7). Pathological analysis revealed disorganized and rough smooth muscle fibers and replacement of the stroma by an increased quantity of collagen fibers (Figures 1-a, 1-b, 1-d, 1-e). Degeneration of the intestinal smooth muscle and increased fibrotic tissue were considered to be the cause of the anorectal stenosis in all the cases. Three patients (Cases 5, 6, 7) had a presacral mass with the fistula communicating the anorectum with the tumor, which histopathological analysis revealed to be lined with columnar or squamous epithelium surrounded by fibrous scar tissue (Figures 1-c, 1-f).

## Discussion

CS is a secondary neural tube dysplasia in which the anterior wall of the spine is formed in the fourth week of embryonic development and the gastrointestinal tract and neural tube are separated. Currarino et al. [2] and several other authors [4,5] suggested that a neuro-enteric fistula is formed in the intestinal and neural tubes, resulting in a presacral mass with a fistula caused by the abnormal splitting or deviation of the notochord during the stage. Gupta et al. [6] also suggested that the failure of some epiblast cells to migrate from their primitive node may leave remnants in the primitive streak, which may develop into a teratoma in the sacrococcygeal region.

A mutation in HLXB9 or MNX1 (formerly HLXB9) in the 7q36 gene has been suggested as the cause of CS. Ross et al. reported that HLXB9 is expressed in the anterior horn of the spinal cord and the lid plate during neural tube development. These abnormalities comprise the so-called “Currarino triad” [7]. However, Dworschak et al. [8] reported that other genes or regulatory regions may contribute to CS, and several cytogenetic studies have implicated further loci in the etiology of CS besides MNX1, based on the finding that mutation analysis detected likely MNX1 variants in only 57.4% of all CS patients. The question of CS etiology, therefore, remains moot.

Because patients with CS present with various, associated malformations and complicated clinical symptoms, an individualized, multifaceted treatment approach is needed [9]. AbouZeid et al. reported 17 cases of CS and described an individualized treatment strategy to address the wide variation in complications [3]. In addition, Martucciello et al. created a diagnostic/therapeutic protocol based on their experience with six patients, but the treatment flow is diverse and complicated [1].

**Figure 2:**
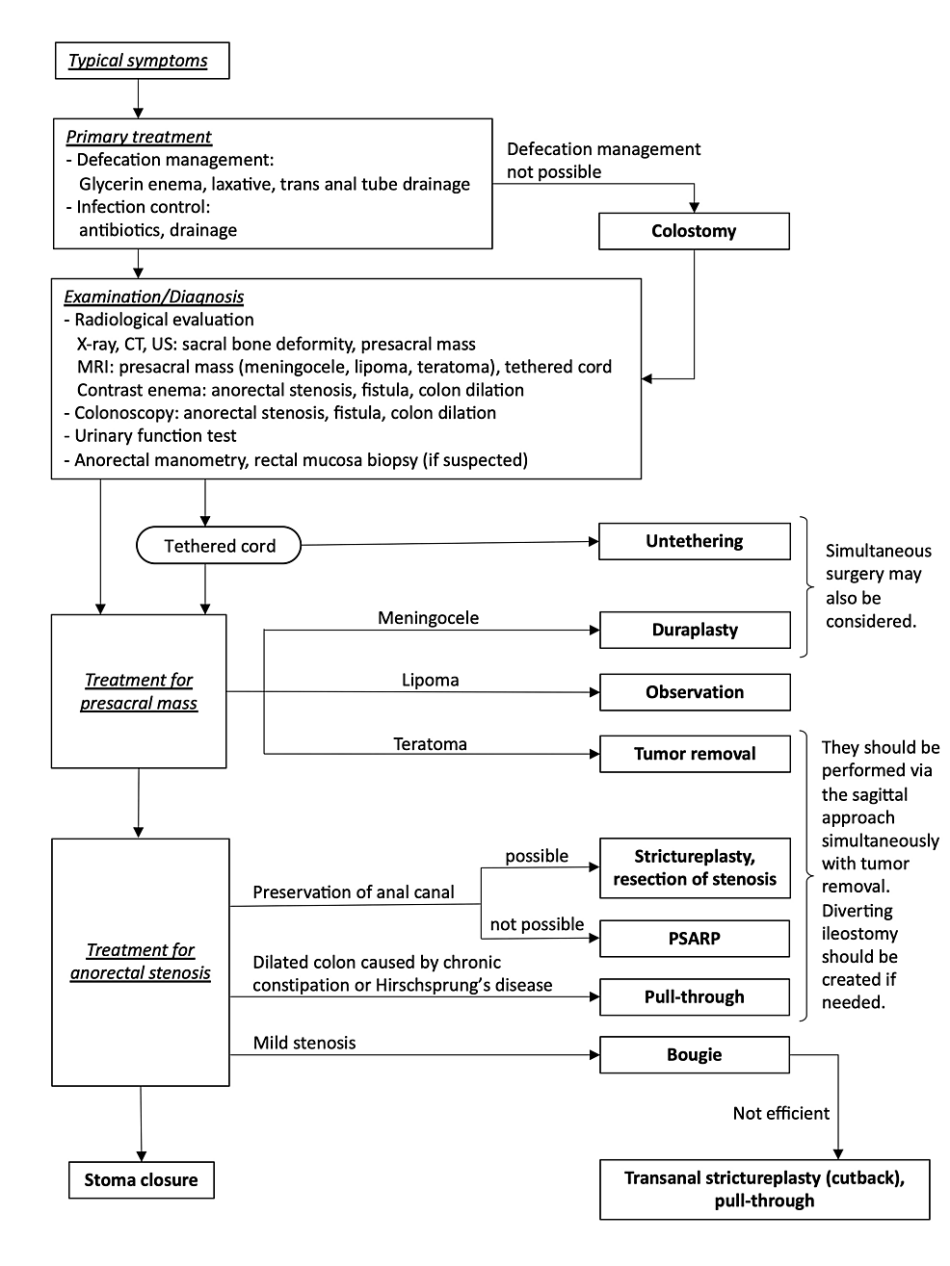
Treatment flowchart for Currarino syndrome with anorectal stenosis.

Anorectal stenosis is the most common type of anorectal malformation in CS, accounting for about 75% of complications [10]. The degree of anorectal stenosis varies from severe, which requires a colostomy in the neonatal period, to mild, which can be relieved by dilation using a metal bougie. Based on our experiences in our seven cases, we created a treatment flow chart for CS with anorectal stenosis (Figure 2).

**Figure 3:**
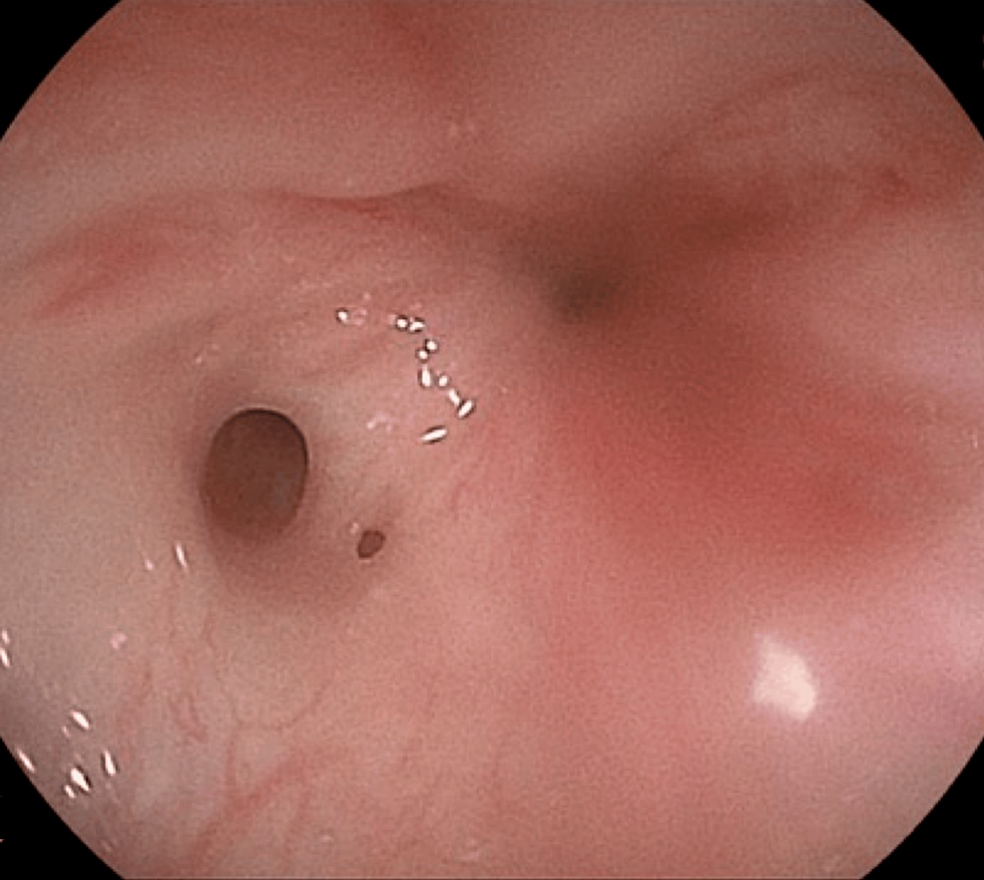
Colonoscopy revealed a fistula communicating between the presacral mass and anorectum in Case 6.

First, the severity of the anorectal stenosis should be assessed. If fecal impaction caused by severe anorectal stenosis is present in the neonatal period, a colostomy should be performed. Defecation in mild-to-moderate cases can be managed by dilation with a metal bougie, glycerin enemas, or a laxative. Next, malformations associated with CS, such as tethered spinal cord and presacral mass, should be assessed using radiological methods, such as magnetic resonance imaging (MRI), computed tomography (CT), or ultrasonography (US). If the presacral mass is infected owing to a fistula communicating with the anorectum, transanal or percutaneous drainage and antibacterial treatment should be performed simultaneously. Contrast enema examination and colonoscopy are also useful to evaluate the degree and extent of anorectal stenosis and may aid in the detection of any fistula communicating the presacral tumor with the anorectum (Figure 3).

**Figure 4:**
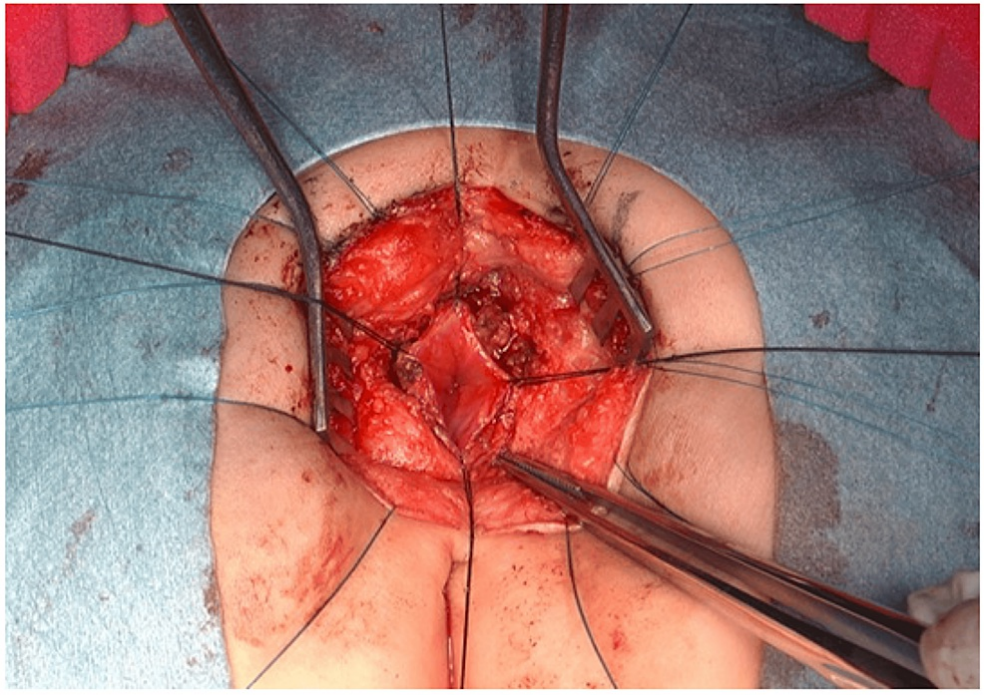
Intra-operative view of strictureplasty for anorectal stenosis in Currarino syndrome. The surgical procedure for anorectal stenosis can be performed simultaneously with presacral mass resection via the same posterior sagittal incision.

Reports of CS complicated with Hirschsprung's disease suggest that anorectal manometry and a rectal mucosal biopsy should also be performed if possible [11,12]. Further, as urinary function can deteriorate in the tethered spinal cord or through neurosurgical complications, a bladder function test should be performed pre- and postoperatively. In Case 1, she was diagnosed by urodynamics, and the intravesical pressure gradually increased from adolescence. A neurogenic bladder can be caused by tethering of the spinal cord, sacral agenesis, recurrence of a presacral mass, or injury to parasympathetic nerves during surgery; however, it was not clear to detect the reason for the neurogenic bladder in our case. If the presacral mass is diagnosed as a teratoma based on radiological findings, a complete surgical resection should be performed to prevent the development of a malignancy or abscess [13]. Determining the surgical indications for anorectal stenosis prior to the removal of the presacral mass is necessary because the surgery for anorectal stenosis can be performed simultaneously with presacral mass resection via the same posterior sagittal incision (Figure 4).

 

Based on pathological analysis of the associated lesion, tumor resection may be insufficient to treat the anorectal stenosis, and surgery at least to the level of the intestinal muscular layer may be needed. Therefore, the procedures for presacral mass excision and stenosis correction should be performed simultaneously if physiological and radiological findings suggest that the stenosis is likely to affect defecation. If the presacral mass is a meningocele, duraplasty and untethering of the tethered spinal cord should be performed first to reduce the risk of meningitis, as the surgical field can become contaminated when the intrarectal lumen is opened to relieve the stenosis in certain procedures.

Whether the surgical procedures for anorectal stenosis and neurosurgery should be sequenced or performed simultaneously is debatable. Given the potential for infection and issues in postoperative management, sequential treatment may be preferable [14]. However, some studies have reported that both procedures can safely be performed simultaneously [15,16].

Various surgical procedures for anorectal stenosis have been reported, and no standard surgical procedure has yet been established. AbouZeid et al. described PSARP for anorectal stenosis in CS to mobilize the rectum in the entire circumference by dividing the muscle complex [2]. On the other hand, Hamrick et al. reported a surgical technique for preserving the anterior anorectal wall by limiting mobilization to the posterior wall half circumference of the anorectal stenosis. This procedure is advantageous for postoperative defecation management because it preserves the sensory nerves near the dentate line [17].

Additional surgery should be considered in patients who experience difficulty with defecation management even after the presacral mass has been removed via the posterior sagittal incision. Given the possibility of postoperative adhesion, using the transanal approach, such as the cutback procedure used in Case 5, which can improve the stenosis without using a posterior sagittal incision, may be preferable. Laparoscopically assisted pull-through surgery has been applied to surgery for anorectal stenosis and is suitable for patients who have previously undergone presacral mass removal [18]. Some patients requiring surgery for segmental dilation of the colon resulting from chronic constipation associated with CS in the distant postoperative period have also been reported [19].

The present case series contained three cases of presacral mass complicated with a fistula between the mass and the anorectum. The histopathological findings of these cases revealed that the location of the anorectal stenosis and the fistula was approximately the same, suggesting that the formation of the presacral mass may be closely related to the anorectal stenosis. Additionally, the anorectal stenosis is more likely to be a congenital, rather than an acquired, abnormality because inflammatory findings were not present in all the cases. The intestinal smooth muscle was disorganized and rough, and the stroma was replaced by an increased quantity of collagen fibers. The fibrous scar tissue may thus lead to anorectal stenosis in CS.

The present case series has several limitations, including its retrospective design and small sample size. Accumulating additional data and undertaking future, multicentric studies with a larger population and a higher level of evidence are required. The surgical benefits for postoperative defecation function were difficult to evaluate fully because three of the patients were too young for evaluation of the postoperative outcomes. In addition, several factors besides anorectal stenosis affect defecation function in CS, such as neurological problems, complications of pelvic surgery, hypoplasia of the anal sphincter and levator ani muscles, and colonic dilation resulting from chronic constipation.

## Conclusions

We reviewed seven patients with CS who underwent treatment in our hospital and proposed a protocol to optimize the treatment flowchart. Since CS has a wide spectrum of clinical presentations, there is no consensus on the therapeutic protocol. Consequently, there is a chance of missing the diagnosis at the initial workup, and optimal treatment could not be given at the appropriate time in some cases. Establishing the treatment strategy could reduce the risk of delayed diagnosis, and it would be possible to improve treatment outcomes based on a full understanding of the complicated pathophysiology of CS.
